# Supplementary material for: Modeling glioblastoma heterogeneity as a dynamic network of cell states
Source: Mol Syst Biol. 2021 Sep 16;17(9):e10105. doi: 10.15252/msb.202010105 (PMC8444284; doi:10.15252/msb.202010105)
Supplement: Supplementary file 5 — Source Data for Figure 3 [file MSB-17-e10105-s001.zip › Figure3A_sourcedata/GSEA_3065/hallmarks_state4.GseaPreranked.1623416296068/HALLMARK_E2F_TARGETS.html]

Details for gene set HALLMARK\_E2F\_TARGETS[GSEA]

|  || Dataset | state4 |
| Phenotype | NoPhenotypeAvailable |
| Upregulated in class | na\_neg |
| GeneSet | HALLMARK\_E2F\_TARGETS |
| Enrichment Score (ES) | -0.81127137 |
| Normalized Enrichment Score (NES) | -3.1042764 |
| Nominal p-value | 0.0 |
| FDR q-value | 0.0 |
| FWER p-Value | 0.0 |
Table: GSEA Results Summary

  

Fig 1: Enrichment plot: HALLMARK\_E2F\_TARGETS      
 Profile of the Running ES Score & Positions of GeneSet Members on the Rank Ordered List

  

| PROBE | GENE SYMBOL | GENE\_TITLE | RANK IN GENE LIST | RANK METRIC SCORE | RUNNING ES | CORE ENRICHMENT || 1 | CDKN1A |  |  | 319 | 0.171 | -0.0480 | No |
| 2 | NAA38 |  |  | 693 | 0.115 | -0.1063 | No |
| 3 | POLE4 |  |  | 1010 | 0.088 | -0.1559 | No |
| 4 | CDKN1B |  |  | 1650 | 0.057 | -0.2592 | No |
| 5 | CDKN2C |  |  | 2822 | 0.028 | -0.4505 | No |
| 6 | NAP1L1 |  |  | 2856 | 0.027 | -0.4552 | No |
| 7 | CDK4 |  |  | 3394 | 0.016 | -0.5429 | No |
| 8 | PPP1R8 |  |  | 3502 | 0.012 | -0.5601 | No |
| 9 | CBX5 |  |  | 3561 | 0.010 | -0.5694 | No |
| 10 | PMS2 |  |  | 3615 | 0.008 | -0.5778 | No |
| 11 | ZW10 |  |  | 4200 | -0.022 | -0.6730 | No |
| 12 | NBN |  |  | 4286 | -0.028 | -0.6863 | No |
| 13 | RFC1 |  |  | 4316 | -0.030 | -0.6903 | No |
| 14 | GSPT1 |  |  | 4383 | -0.034 | -0.7002 | No |
| 15 | XRCC6 |  |  | 4437 | -0.039 | -0.7080 | No |
| 16 | TFRC |  |  | 4532 | -0.047 | -0.7222 | No |
| 17 | MYC |  |  | 4618 | -0.053 | -0.7348 | No |
| 18 | POLD2 |  |  | 4714 | -0.061 | -0.7489 | No |
| 19 | SHMT1 |  |  | 4824 | -0.069 | -0.7650 | No |
| 20 | POLD1 |  |  | 4853 | -0.072 | -0.7678 | No |
| 21 | IPO7 |  |  | 4897 | -0.075 | -0.7730 | No |
| 22 | SYNCRIP |  |  | 4908 | -0.075 | -0.7727 | No |
| 23 | EED |  |  | 4990 | -0.082 | -0.7840 | No |
| 24 | WDR90 |  |  | 5018 | -0.084 | -0.7863 | No |
| 25 | DONSON |  |  | 5051 | -0.087 | -0.7894 | No |
| 26 | NUP205 |  |  | 5053 | -0.087 | -0.7874 | No |
| 27 | DCK |  |  | 5068 | -0.088 | -0.7874 | No |
| 28 | NME1 |  |  | 5086 | -0.090 | -0.7880 | No |
| 29 | TIPIN |  |  | 5131 | -0.095 | -0.7928 | No |
| 30 | DSCC1 |  |  | 5133 | -0.095 | -0.7906 | No |
| 31 | JPT1 |  |  | 5145 | -0.096 | -0.7900 | No |
| 32 | UNG |  |  | 5148 | -0.096 | -0.7879 | No |
| 33 | SMC6 |  |  | 5149 | -0.096 | -0.7855 | No |
| 34 | CDC25A |  |  | 5172 | -0.099 | -0.7867 | No |
| 35 | PLK4 |  |  | 5210 | -0.102 | -0.7902 | No |
| 36 | CCNE1 |  |  | 5218 | -0.103 | -0.7888 | No |
| 37 | RNASEH2A |  |  | 5241 | -0.105 | -0.7897 | No |
| 38 | MTHFD2 |  |  | 5256 | -0.107 | -0.7894 | No |
| 39 | POP7 |  |  | 5259 | -0.107 | -0.7870 | No |
| 40 | SRSF1 |  |  | 5281 | -0.108 | -0.7878 | No |
| 41 | UBR7 |  |  | 5340 | -0.112 | -0.7945 | No |
| 42 | RPA2 |  |  | 5372 | -0.115 | -0.7967 | No |
| 43 | RFC3 |  |  | 5450 | -0.124 | -0.8062 | No |
| 44 | TUBB |  |  | 5482 | -0.129 | -0.8080 | Yes |
| 45 | RAD21 |  |  | 5496 | -0.131 | -0.8069 | Yes |
| 46 | MRE11 |  |  | 5507 | -0.132 | -0.8052 | Yes |
| 47 | RAD51C |  |  | 5525 | -0.134 | -0.8047 | Yes |
| 48 | GINS1 |  |  | 5542 | -0.135 | -0.8039 | Yes |
| 49 | RBBP7 |  |  | 5546 | -0.136 | -0.8010 | Yes |
| 50 | HMGB3 |  |  | 5584 | -0.142 | -0.8035 | Yes |
| 51 | DCTPP1 |  |  | 5588 | -0.143 | -0.8005 | Yes |
| 52 | PSIP1 |  |  | 5590 | -0.143 | -0.7970 | Yes |
| 53 | CSE1L |  |  | 5597 | -0.144 | -0.7944 | Yes |
| 54 | POLA2 |  |  | 5615 | -0.146 | -0.7936 | Yes |
| 55 | POLE |  |  | 5624 | -0.147 | -0.7912 | Yes |
| 56 | CTPS1 |  |  | 5635 | -0.150 | -0.7891 | Yes |
| 57 | CDCA8 |  |  | 5642 | -0.151 | -0.7863 | Yes |
| 58 | MCM6 |  |  | 5644 | -0.151 | -0.7827 | Yes |
| 59 | TRA2B |  |  | 5652 | -0.152 | -0.7800 | Yes |
| 60 | TUBG1 |  |  | 5690 | -0.158 | -0.7821 | Yes |
| 61 | HNRNPD |  |  | 5705 | -0.161 | -0.7804 | Yes |
| 62 | MSH2 |  |  | 5711 | -0.162 | -0.7772 | Yes |
| 63 | E2F8 |  |  | 5717 | -0.163 | -0.7739 | Yes |
| 64 | ILF3 |  |  | 5722 | -0.165 | -0.7704 | Yes |
| 65 | PRIM2 |  |  | 5736 | -0.169 | -0.7684 | Yes |
| 66 | SLBP |  |  | 5748 | -0.172 | -0.7659 | Yes |
| 67 | EXOSC8 |  |  | 5751 | -0.173 | -0.7619 | Yes |
| 68 | KIF18B |  |  | 5760 | -0.174 | -0.7588 | Yes |
| 69 | NCAPD2 |  |  | 5778 | -0.179 | -0.7572 | Yes |
| 70 | BARD1 |  |  | 5779 | -0.179 | -0.7527 | Yes |
| 71 | MCM2 |  |  | 5785 | -0.180 | -0.7490 | Yes |
| 72 | TRIP13 |  |  | 5792 | -0.181 | -0.7455 | Yes |
| 73 | TCF19 |  |  | 5796 | -0.182 | -0.7414 | Yes |
| 74 | TIMELESS |  |  | 5832 | -0.190 | -0.7424 | Yes |
| 75 | PLK1 |  |  | 5836 | -0.192 | -0.7381 | Yes |
| 76 | PA2G4 |  |  | 5841 | -0.193 | -0.7339 | Yes |
| 77 | PAICS |  |  | 5858 | -0.196 | -0.7317 | Yes |
| 78 | CIT |  |  | 5864 | -0.198 | -0.7275 | Yes |
| 79 | DIAPH3 |  |  | 5893 | -0.205 | -0.7270 | Yes |
| 80 | SPAG5 |  |  | 5899 | -0.207 | -0.7227 | Yes |
| 81 | BUB1B |  |  | 5901 | -0.207 | -0.7177 | Yes |
| 82 | WEE1 |  |  | 5907 | -0.209 | -0.7133 | Yes |
| 83 | SNRPB |  |  | 5908 | -0.209 | -0.7080 | Yes |
| 84 | BRCA2 |  |  | 5910 | -0.210 | -0.7030 | Yes |
| 85 | NOP56 |  |  | 5921 | -0.213 | -0.6993 | Yes |
| 86 | MMS22L |  |  | 5928 | -0.215 | -0.6949 | Yes |
| 87 | RANBP1 |  |  | 5939 | -0.218 | -0.6911 | Yes |
| 88 | NUP107 |  |  | 5949 | -0.220 | -0.6871 | Yes |
| 89 | PSMC3IP |  |  | 5951 | -0.222 | -0.6817 | Yes |
| 90 | RACGAP1 |  |  | 5953 | -0.223 | -0.6763 | Yes |
| 91 | LUC7L3 |  |  | 5954 | -0.224 | -0.6707 | Yes |
| 92 | POLD3 |  |  | 5957 | -0.225 | -0.6654 | Yes |
| 93 | XPO1 |  |  | 5964 | -0.227 | -0.6607 | Yes |
| 94 | USP1 |  |  | 5986 | -0.233 | -0.6583 | Yes |
| 95 | RAN |  |  | 5990 | -0.235 | -0.6529 | Yes |
| 96 | STMN1 |  |  | 5993 | -0.236 | -0.6474 | Yes |
| 97 | KIF2C |  |  | 5996 | -0.237 | -0.6418 | Yes |
| 98 | SPC24 |  |  | 6000 | -0.238 | -0.6363 | Yes |
| 99 | LIG1 |  |  | 6002 | -0.239 | -0.6305 | Yes |
| 100 | RPA3 |  |  | 6014 | -0.244 | -0.6262 | Yes |
| 101 | SMC1A |  |  | 6017 | -0.245 | -0.6204 | Yes |
| 102 | CHEK1 |  |  | 6025 | -0.248 | -0.6154 | Yes |
| 103 | AURKA |  |  | 6026 | -0.248 | -0.6092 | Yes |
| 104 | DEPDC1 |  |  | 6030 | -0.251 | -0.6034 | Yes |
| 105 | PNN |  |  | 6040 | -0.265 | -0.5982 | Yes |
| 106 | SMC3 |  |  | 6041 | -0.265 | -0.5916 | Yes |
| 107 | MXD3 |  |  | 6042 | -0.265 | -0.5850 | Yes |
| 108 | GINS4 |  |  | 6048 | -0.267 | -0.5791 | Yes |
| 109 | MCM5 |  |  | 6069 | -0.278 | -0.5755 | Yes |
| 110 | SPC25 |  |  | 6072 | -0.279 | -0.5688 | Yes |
| 111 | KIF22 |  |  | 6089 | -0.290 | -0.5642 | Yes |
| 112 | DEK |  |  | 6092 | -0.292 | -0.5572 | Yes |
| 113 | RFC2 |  |  | 6095 | -0.294 | -0.5502 | Yes |
| 114 | DUT |  |  | 6107 | -0.303 | -0.5445 | Yes |
| 115 | MCM3 |  |  | 6113 | -0.307 | -0.5376 | Yes |
| 116 | MELK |  |  | 6117 | -0.312 | -0.5303 | Yes |
| 117 | LBR |  |  | 6118 | -0.312 | -0.5225 | Yes |
| 118 | ASF1B |  |  | 6127 | -0.320 | -0.5158 | Yes |
| 119 | SRSF2 |  |  | 6128 | -0.321 | -0.5078 | Yes |
| 120 | HMGA1 |  |  | 6132 | -0.324 | -0.5002 | Yes |
| 121 | LMNB1 |  |  | 6134 | -0.325 | -0.4923 | Yes |
| 122 | DDX39A |  |  | 6137 | -0.328 | -0.4844 | Yes |
| 123 | TACC3 |  |  | 6138 | -0.328 | -0.4762 | Yes |
| 124 | CCNB2 |  |  | 6140 | -0.329 | -0.4681 | Yes |
| 125 | AURKB |  |  | 6143 | -0.332 | -0.4601 | Yes |
| 126 | TK1 |  |  | 6144 | -0.334 | -0.4518 | Yes |
| 127 | HMMR |  |  | 6148 | -0.340 | -0.4438 | Yes |
| 128 | PRKDC |  |  | 6149 | -0.341 | -0.4353 | Yes |
| 129 | CDC20 |  |  | 6154 | -0.344 | -0.4274 | Yes |
| 130 | PRDX4 |  |  | 6155 | -0.345 | -0.4187 | Yes |
| 131 | DLGAP5 |  |  | 6156 | -0.345 | -0.4101 | Yes |
| 132 | EZH2 |  |  | 6159 | -0.348 | -0.4018 | Yes |
| 133 | CDKN3 |  |  | 6160 | -0.349 | -0.3930 | Yes |
| 134 | KIF4A |  |  | 6164 | -0.355 | -0.3847 | Yes |
| 135 | CDCA3 |  |  | 6165 | -0.355 | -0.3758 | Yes |
| 136 | RAD51AP1 |  |  | 6167 | -0.357 | -0.3670 | Yes |
| 137 | ANP32E |  |  | 6168 | -0.357 | -0.3581 | Yes |
| 138 | MCM4 |  |  | 6171 | -0.372 | -0.3491 | Yes |
| 139 | PCNA |  |  | 6173 | -0.374 | -0.3400 | Yes |
| 140 | CENPM |  |  | 6179 | -0.386 | -0.3312 | Yes |
| 141 | DNMT1 |  |  | 6182 | -0.396 | -0.3216 | Yes |
| 142 | NASP |  |  | 6187 | -0.403 | -0.3122 | Yes |
| 143 | MYBL2 |  |  | 6190 | -0.408 | -0.3023 | Yes |
| 144 | BRCA1 |  |  | 6197 | -0.435 | -0.2924 | Yes |
| 145 | RRM2 |  |  | 6199 | -0.438 | -0.2816 | Yes |
| 146 | BIRC5 |  |  | 6214 | -0.484 | -0.2718 | Yes |
| 147 | SMC4 |  |  | 6219 | -0.490 | -0.2602 | Yes |
| 148 | HELLS |  |  | 6221 | -0.501 | -0.2479 | Yes |
| 149 | CENPE |  |  | 6226 | -0.511 | -0.2358 | Yes |
| 150 | TMPO |  |  | 6233 | -0.522 | -0.2237 | Yes |
| 151 | CKS1B |  |  | 6234 | -0.523 | -0.2106 | Yes |
| 152 | KPNA2 |  |  | 6236 | -0.543 | -0.1972 | Yes |
| 153 | ORC6 |  |  | 6238 | -0.585 | -0.1828 | Yes |
| 154 | CKS2 |  |  | 6239 | -0.585 | -0.1681 | Yes |
| 155 | UBE2T |  |  | 6240 | -0.593 | -0.1533 | Yes |
| 156 | ATAD2 |  |  | 6243 | -0.607 | -0.1385 | Yes |
| 157 | UBE2S |  |  | 6244 | -0.617 | -0.1231 | Yes |
| 158 | PTTG1 |  |  | 6245 | -0.622 | -0.1075 | Yes |
| 159 | CDK1 |  |  | 6246 | -0.627 | -0.0919 | Yes |
| 160 | MAD2L1 |  |  | 6247 | -0.628 | -0.0762 | Yes |
| 161 | MCM7 |  |  | 6252 | -0.665 | -0.0602 | Yes |
| 162 | HMGB2 |  |  | 6258 | -0.766 | -0.0419 | Yes |
| 163 | TOP2A |  |  | 6260 | -0.814 | -0.0217 | Yes |
| 164 | MKI67 |  |  | 6261 | -0.882 | 0.0003 | Yes |
Table: GSEA details [plain text format]

  

Fig 2: HALLMARK\_E2F\_TARGETS: Random ES distribution      
 Gene set null distribution of ES for **HALLMARK\_E2F\_TARGETS**
